# Complex Regional Pain Syndrome Type I Affects Brain Structure in Prefrontal and Motor Cortex

**DOI:** 10.1371/journal.pone.0085372

**Published:** 2014-01-09

**Authors:** Burkhard Pleger, Bogdan Draganski, Peter Schwenkreis, Melanie Lenz, Volkmar Nicolas, Christoph Maier, Martin Tegenthoff

**Affiliations:** 1 Department of Neurology, Max Planck Institute for Human Cognitive and Brain Sciences, Leipzig, Germany; 2 Clinic for Cognitive Neurology, University Hospital Leipzig, Leipzig, Germany; 3 Laboratoire de Recherche en Neuroimagerie – LREN, Departement des neurosciences cliniques, Centre Hospitalier Universitaire Vaudois (CHUV), Lausanne, Switzerland; 4 Department of Neurology, University Hospital Bergmannsheil, Bochum, Germany; 5 Department of Radiology, University Hospital Bergmannsheil, Bochum, Germany; 6 Department of Pain Treatment, University Hospital Bergmannsheil, Bochum, Germany; Hangzhou Normal University, China

## Abstract

The complex regional pain syndrome (CRPS) is a rare but debilitating pain disorder that mostly occurs after injuries to the upper limb. A number of studies indicated altered brain function in CRPS, whereas possible influences on brain structure remain poorly investigated.

We acquired structural magnetic resonance imaging data from CRPS type I patients and applied voxel-by-voxel statistics to compare white and gray matter brain segments of CRPS patients with matched controls. Patients and controls were statistically compared in two different ways: First, we applied a 2-sample ttest to compare whole brain white and gray matter structure between patients and controls. Second, we aimed to assess structural alterations specifically of the primary somatosensory (S1) and motor cortex (M1) contralateral to the CRPS affected side. To this end, MRI scans of patients with left-sided CRPS (and matched controls) were horizontally flipped before preprocessing and region-of-interest-based group comparison. The unpaired ttest of the “non-flipped” data revealed that CRPS patients presented increased gray matter density in the dorsomedial prefrontal cortex. The same test applied to the “flipped” data showed further increases in gray matter density, not in the S1, but in the M1 contralateral to the CRPS-affected limb which were inversely related to decreased white matter density of the internal capsule within the ipsilateral brain hemisphere. The gray-white matter interaction between motor cortex and internal capsule suggests compensatory mechanisms within the central motor system possibly due to motor dysfunction. Altered gray matter structure in dorsomedial prefrontal cortex may occur in response to emotional processes such as pain-related suffering or elevated analgesic top-down control.

## Introduction

The complex regional pain syndrome (CRPS) is a rare but strongly debilitating pain disorder that mostly occurs after upper limb injuries. Severe pain is the most prominent symptom, which is disproportionally strong with respect to the inciting trauma. Clinically, CRPS is subdivided into two types: type I, formerly known as reflex sympathetic dystrophy, Sudeck's atrophy, reflex neurovascular dystrophy, or algoneurodystrophy, occurs without, and type II, formerly known as causalgia, with peripheral nerve damage [1]. The pain has a neuropathic character, is not restricted to certain dermatomes and is accompanied by a number of symptoms like abnormal sudomotor activity, swelling of the affected limb, changing skin color, temperature change, as well as an impaired hair and nail growth [2], [3].

Although CRPS is well classified and treatment improved over the last decade, the inciting pathophysiological mechanisms are still a matter of ongoing research. Both peripheral and central nervous system mechanisms seem to be involved. These include peripheral and central sensitization, inflammation [4], genetic factors [5], and disturbances of the sympathetic nervous system [6]–[8]. Clinical observations confirmed numbness when testing for somatosensory impairments which were not restricted to the affected limb but covered half of the body on the CRPS-affected side [9], [10]. Such a clinical picture reminds of a stroke-like deficit indicating a central nervous origin. Non-invasive functional brain imaging and electrophysiological experiments agree on this notion, suggesting shrinkage of the cortical representation of the affected limb in primary sensorimotor cortex [11]–[14] and disinhibition of motor and somatosensory cortex excitability [15], [16]. Together these findings point to complex dysfunctions of central sensorimotor representations [17]–[19].

Besides these convincing evidences pointing to functional alterations of the central nervous system in CRPS, changes in brain structure remain poorly investigated. One recent magnetic resonance imaging (MRI) study systematically investigated gray and white matter morphology in CRPS patients as compared to matched controls. The findings suggest that chronic CRPS is related to alterations of those brain structures involved in emotional, autonomic, and pain perception [20].

In the present structural MRI study we investigated CRPS associated alterations in two different ways. First, we applied voxel-by-voxel statistics to compare the global gray and white matter structures of CRPS patients to age and gender matched controls. Second, we specifically investigated structural alterations of the cortical representations of the affected limb in the contralateral primary somatosensory cortex (S1), located on the postcentral gyrus, and the primary motor cortex (M1), located on the precentral gyrus, to address the question whether the functional alterations, as described in previous functional brain imaging studies [11]–[16], consolidate to brain structure. To increase statistical power, we horizontally flipped MRI scans of left-sided CRPS patients and associated controls before preprocessing and applied region-of-interest based statistics. To investigate structural alterations in the S1 and the M1 specifically related to CRPS and not to any peripheral nerve damages, we included only patients suffering from CRPS type I (i.e., without a peripheral nerve damage).

## Methods

### CRPS patients and control subjects

We included 20 patients with CRPS type I (11 females, mean age 41.8 +/– 9.8 years, for further information on inciting trauma, CRPS duration, current pain, and pain experienced over the last month see Table 1). Twenty age and gender matched healthy individuals served as controls (11 females, mean age 41.6 +/– 9.6 years). The study was approved by the local ethics committee of the Ruhr-University of Bochum and all patients and control subjects gave written informed consent. Before inclusion, patients first underwent electroneurographic and clinical neurological examination to exclude a peripheral nerve injury (i.e., CRPS type II) as another possible source of cortical reorganization [21]. Only patients in whom signs of CRPS affected the whole hand including all digits were recruited. In all patients, we found an increased bone metabolism of the affected hand as shown by three-phase scintigraphy [22].

**Table 1:** Clinical features of CRPS patients.

Patient No.	Inciting trauma	CRPS affected side	Age (years)	CRPS since (months)	Current pain (NRS)	Pain over last month (NRS)
#1	Fracture of radius	Left	52	10	3	3
#2	Fracture of humerus body	Left	39	1	8	4
#3	Fracture of radius	Right	44	3	1.5	4
#4	Strain trauma	Left	52	9	4	4
#5	Fracture of radius	Right	50	5	6	4
*#6*	Bruise of radius head	Left	34	7	8	4
#7	Fracture of metacarpus	Right	22	17	3	4
#8	Fracture of os naviculare	Right	53	16	8	6
#9	Contusion of thumb	Right	48	13	7	5
#10	Fracture of radius	Right	40	14	7	3
#11	Fracture of radius	Left	53	3	7	5
#12	Fracture of radius	Right	27	63	1	5
#13	Contusion of hand	Right	30	4	6	5
#14	Fracture of radius	Left	49	4	6	5
#15	Bruise of radius head	Right	42	4	1.5	2
#16	Fracture of metacarpus, digit II and III	Left	49	5	2	4
#17	Complex fracture of forearm	Right	28	8	5	4
#18	Fracture of radius	Left	40	12	7	5
#19	Fracture of metacarpus	Right	34	5	7	5
#20	Cutting injury	Right	50	36	8	6

### MRI data acquisition

Structural MRI measurement was performed with a whole body 1.5 T MRI scanner (Magnetom Symphony, Siemens Medical Systems, Germany). During MRI scanning, the head of participants was placed in a standard imaging head coil. Anatomical T1-weighted scans were acquired using an isotropic T1-3dGE (MPRAGE) sequence (TI 1100 ms, TR 1790 ms, TE 3.87 ms, matrix 256 * 256, FOV 256 mm, flip angle 15-, 1-mm slice thickness, no gap, voxel size: 1 * 1 * 1 mm) with 160 sagittal orientated slices covering the whole brain.

### MRI data pre-processing

We used SPM 8 (Wellcome Trust Centre for Neuroimaging, University College London, London, UK; http://www.fil.ion.ucl.ac.uk/spm) and the voxel-based morphometry (VBM) toolbox 8 (http://dbm.neuro.uni-jena.de/vbm.html) implemented in Matlab (Mathworks, version 7.9) for pre- and post-processing of T1-weighted images. Images were bias corrected, segmented, and registered (using rigid-body transformation with translation and rotation about the three axes) to standardized Montreal Neurological Institute (MNI) space using the unified segmentation approach [23]. To account for local compression and expansion during transformation, gray matter (GM) and white matter (WM) segments were scaled by the Jacobian determinants of the deformations (i.e., modulation). The Jacobian determinants were defined from non-linear registration, but without accounting for the scale factor from affine registration. This procedure produces tissue class images in alignment with the template, but multiplies the voxel values by the non-linear components. This applies the correction for numeric brain volume directly to the data. Finally, we applied a Gaussian smoothing kernel of 7 mm full width at half maximum (FWHM) to the modulated GM and WM volumes. We here applied a small smoothing kernel that matches the assumed anatomical sizes of the regions of interest [24].

For statistical inference, we discarded all voxels with a GM value below 0.2 to avoid possible partial volume effects near the border to WM.

### Whole brain group analyses of gray and white matter structure

To assess global GM and WM changes in CRPS, we compared patients and matched control subjects using a two-sample ttest as implemented in SPM 8. Age and gender were included as nuisance variables. We tested for increases and decreases of GM and WM in CRPS patients as compared to healthy controls. Clusters of voxels were considered significant if the voxel level exceeded a threshold of *p = *0.001 (uncorrected) and the cluster size a threshold of *p = *0.05, family-wise error (FWE) corrected for multiple comparisons with respect to the Gaussian random field theory [25].

### Region-of-interest group analysis to assess structural alteration on the postcentral (S1) and the precentral gyrus (M1)

For the investigation of structural changes of the CRPS affected limb’s representation in the S1 and the M1 contralateral to the CRPS affected side, T1-weighted MRI scans of patients with left-sided CRPS (and their individual controls) were left-to-right flipped before pre-processing. The next processing steps were the same as described above in the previous paragraphs. According to our [13]–[16] and others previous observations [11], [12] we expected structural changes of the representation of the CRPS affected upper limb in the S1 and the M1. We thus restricted our search volume to either the precentral (anatomical site of the M1) or the postcentral gyrus (anatomical site of the S1) using the corresponding ROIs of the aal atlas [26], as implemented in the WFU PickAtlas toolbox for SPM [27]. Significant clusters with a voxel threshold of *p = *0.001 (uncorrected) and a cluster size threshold of *p = *0.05, family-wise error (FWE) within the anatomical ROIs were considered significant.

### Correlation between gray and white matter structures and between brain structures and clinical features

Next, we computed the mean across significant voxels within the clusters of GM and WM for each patient and each analysis. These values were implemented in a Pearson correlation analysis to test for a positive or negative correlation with the clinical features (i.e., CRPS duration, current pain, pain experienced over the last month) and between GM and WM structures.

Besides the Pearson correlation we used to identify a possible relationship between clinical data and GM/WM effects, we also tested for a relationship between each clinical feature and all voxels throughout patients’ WM and GM segments. To this end, we used the regression analysis as implemented in SPM 8. Each statistical model consisted of patients’ GM or WM segments and one regressor representing one clinical feature (i.e., 6 regression analyses: 2 brain structure segments: WM and GM; 3 clinical features: CRPS duration, current pain, pain experienced over the last month). Age and gender were included as nuisance variables.

## Results

### CRPS patients

All patients fulfilled the Budapest clinical diagnostic criteria for CRPS (see [3]; clinical signs of patients as listed in Table 1: allodynia: #1, #8, #10, #13, #14, #18–#20; temperature asymmetry: #1, #4, #5, #15, #19; skin color changes: #1–#20; skin color asymmetry: #1–#20; edema: none; sweating changes: #2, #5, #14, #18; sweating asymmetry: #2, #5, #14, #18; decreased range of motion: #1–#20; motor dysfunctions: #1–#20; trophic changes (hair, nail, skin): #1–#20; hyperalgesia to pinprick: #1, #3–#20). All patients estimated their pain intensity experienced during the last month as well as the pain intensity felt directly before the MRI session on a numeric rating scale (NRS: ranging from 0 = no pain to 10 = most severe pain).

### Whole brain group analyses of gray matter structure

**Figure 1:**
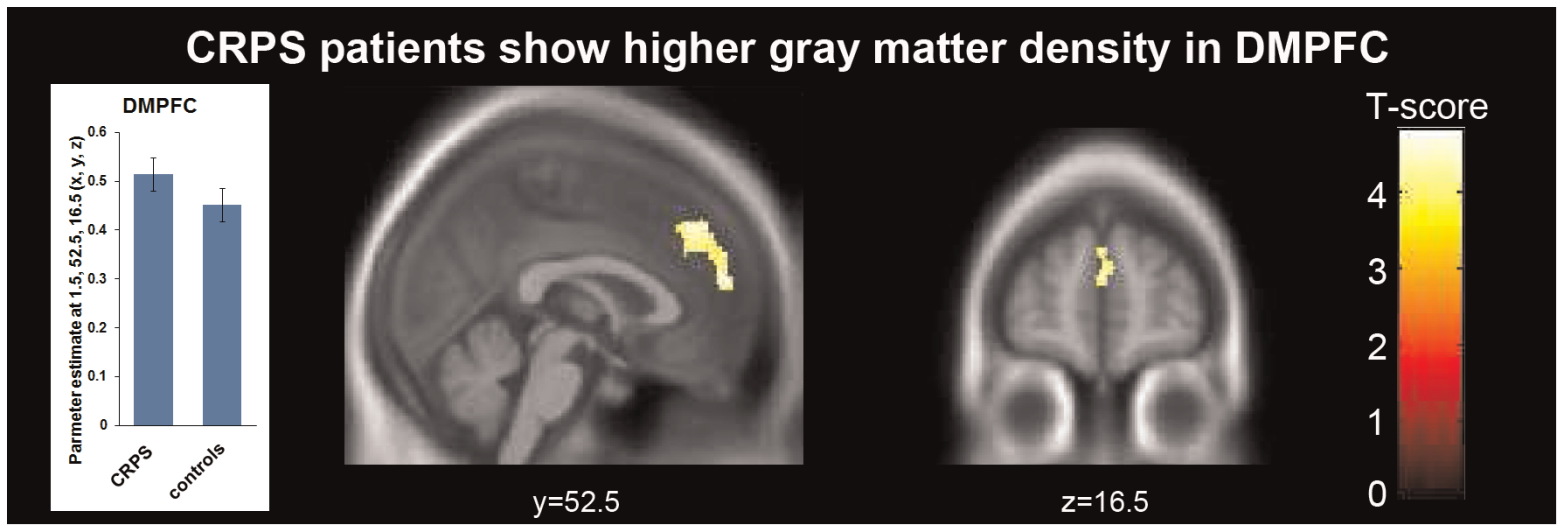
Comparing CRPS patients to age and gender matched controls by VBM whole brain analysis identified one significant cluster with increased gray matter density in CRPS patients located in the DMPFC (peak voxel-level: 1.5, 52.5, 16.5 (x, y, z, mm), T = 4.42, p<0.001 uncorrected, cluster-level: 556 voxels, p = 0.03 FWE corrected). Since we found no link to the individual clinical CRPS features (see Results), the interpretation of this finding remains speculative, but it may point to pain associated altered cognitive processes such as emotional suffering. The bar plot represents estimated gray matter density from the peak voxel. The whiskers indicate the standard error.

The two-sample ttest on the GM segments revealed an increased GM density in CRPS patients only in one cluster which was localized in the dorsomedial prefrontal cortex (DMPFC, peak voxel-level: 1.5, 52.5, 16.5 (x, y, z, mm), T = 4.42, p<0.001 uncorrected, cluster-level: 556 voxels, p = 0.03 FWE corrected, see Fig. 1 and Table 2). No other significant clusters were found throughout the gray matter segment that indicated an increase or decrease in GM density in CRPS patients as compared to controls.

**Table 2:** Overview on gray and white matter findings when comparing CRPS patients to age and gender matched healthy control subjects (DMPFC: dorsomedial prefrontal cortex; M1: primary motor cortex; S1: primary somatosensory cortex).

	Peak voxel-level			Cluster-level	
Brain region	MNI coordinates (x, y, z in mm)	T-score	p-value (uncorrected)	voxels	p-value (corrected)
DMPFC	1.5, 52.5, 16.5	4.42	p<0.001	556	0.03
M1	–40.5, –9, 60	4.31	p<0.001	89	0.042
S1	-	-	-	-	-
Internal capsule	–21, 3, 4.5	4.07	p<0.001	789	0.014

### Region-of-interest group analysis of structural alteration on the postcentral (S1) and the precentral gyrus (M1)

**Figure 2:**
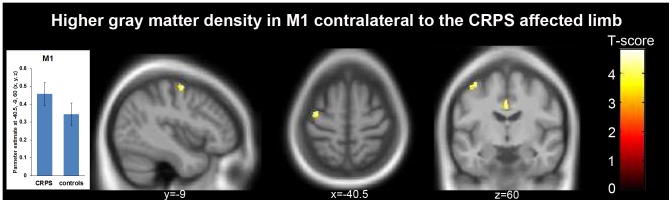
To identify structural changes in the somatosensory cortex, MRI scans of patients with left-sided CRPS were horizontally flipped before data pre-processing and statistical comparison (i.e., CRPS > controls). Using small volume correction we found one significant cluster with increased gray matter density, which was located in the primary motor cortex (M1: peak voxel-level: –40.5, –9, 60 (x, y, z, mm), T = 4.31, p<0.001 uncorrected, cluster-level: 89 voxels, p = 0.042 FWE corrected). As for the DMPFC (see Fig. 1), we found no correlation between altered grey matter density in primary motor cortex and clinical CRPS features (see Results). The bar plot shows estimated gray matter density of the peak voxel and whiskers indicate standard error.

The region-of-interest based analysis of voxels within the postcentral gyrus revealed no significant clusters of altered GM density (see Methods for further details on ROI definition). On the precentral gyrus we however found one cluster of significantly altered GM density (M1, peak voxel-level: –40.5, –9, 60 (x, y, z, mm), T = 4.31, p<0.001 uncorrected, cluster-level: 89 voxels, p = 0.042 FWE corrected, see Fig. 2 and Table 2) contralateral to the affected upper limb, indicating and increase in GM density in the M1. We found no other significant increases or decreases in GM density.

### Whole brain group analysis of white matter structure

**Figure 3:**
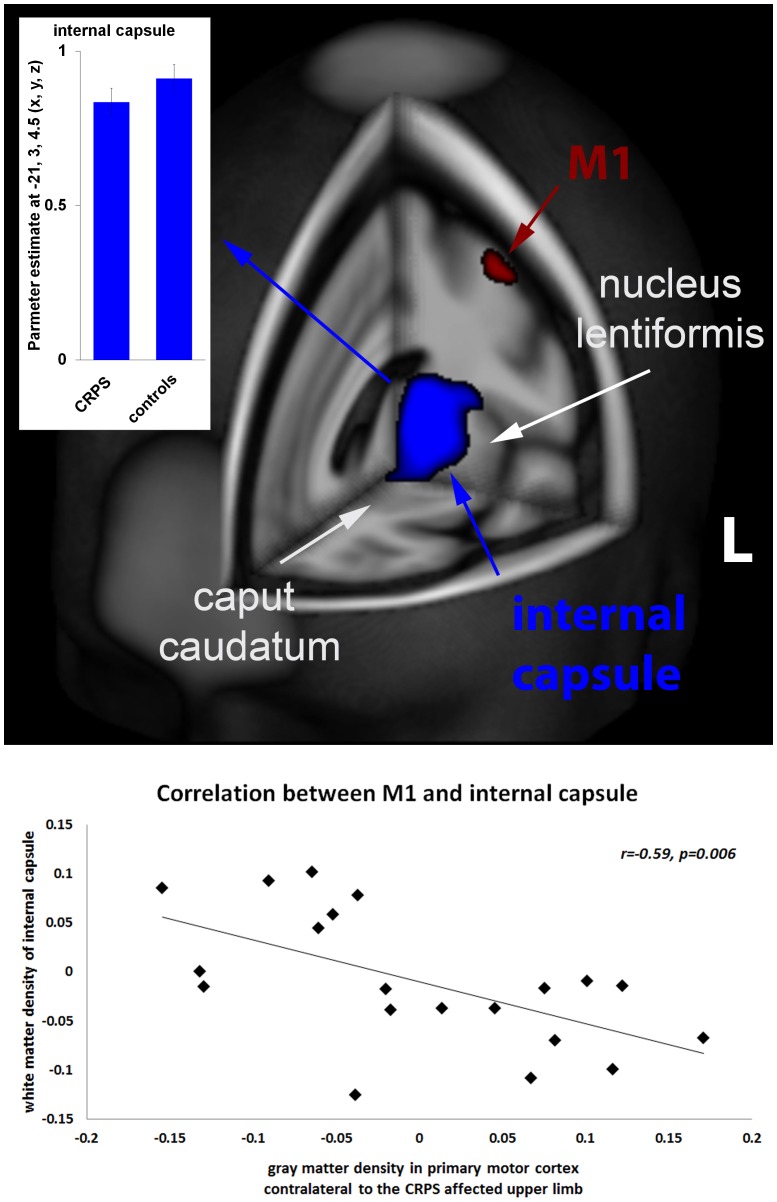
Voxel-wise VBM statistics of whole brain white matter structure revealed one significant cluster with decreased white matter density when comparing CRPS patients to matched controls (see blue cluster, peak voxel-level:–21, 3, 4.5 (x, y, z, mm), T = 4.07, p<0.001 uncorrected, cluster-level: 789 voxels, p = 0.014 FWE corrected). This cluster was located in the internal capsule, known to contain the projections from the M1 to the brainstem. In agreement with this anatomical implementation, we found that reduced individual white matter density negatively correlated (see scatter plot) with increased gray matter density we found in primary motor cortex (M1, see red cluster and Fig. 2). The red diamonds indicate patients with left-sided CRPS. Bar plot indicates estimated gray matter changes of the peak voxel; whiskers the standard error.

The two-sample ttest on “flipped” and “non-flipped” WM segments revealed only one significant cluster that was located in the internal capsule (IC). The cluster location, extend, and significance level for both ttest were almost the same. For redundancy reasons we therefore only report the “flipped” results on the IC: peak voxel-level: –21, 3, 4.5 (x, y, z, mm), T = 4.07, p<0.001 uncorrected, cluster-level: 789 voxels, p = 0.014 FWE corrected, see Fig. 3 and Table 2. These findings indicate a deceased in WM density contralateral to the CRPS affected limb.

### Correlation between gray and white matter structures and between brain structures and clinical features

**Figure 4:**
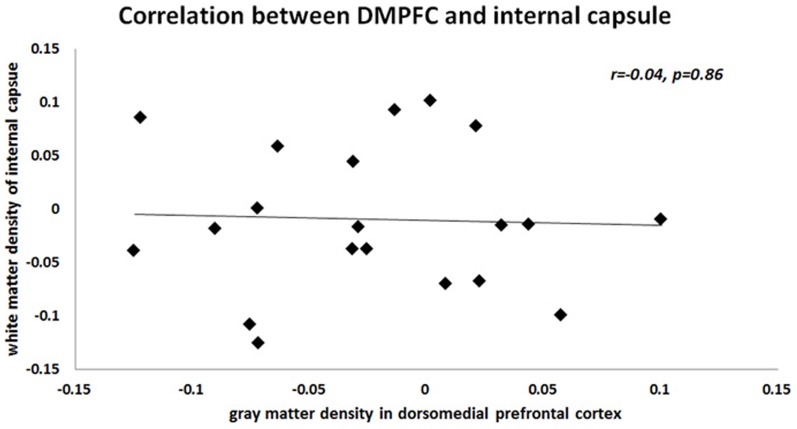
Unlike the correlation we found between gray matter structure in the M1 and the internal capsule, we found no correlation with gray matter density in the DMPFC.

Next, we computed the mean across voxels within significant GM (i.e., DMPFC and M1) and WM (i.e., IC) clusters for all patients and applied these to Pearson’s correlation analyses to test for a possible relationship with the individual clinical features, as well as between GM and WM densities. We found no relationship between any significant GM and WM clusters and clinical features (current pain intensity, DMPFC: r = 0.08, M1: r = 0.06, IC: r = –0.22; pain experienced over last month, DMPFC: r = –0.03, M1: r = –0.19, IC: r = –0.12; duration of CRPS, DMPFC: r = –0.05, M1: r = –0.27, IC: r = 0.29). We also found no significant correlation between GM density in the DMPFC and WM density in the IC (see Fig. 4), but a significant negative correlation between GM density in the M1 and WM density in the IC (see Fig. 3). This negative correlation suggests that the higher the GM density in the M1 the lower the WM density in the ipsilateral IC. As for the two-sample ttest (see above) results of the correlation analyses for “non-flipped” WM densities almost matched the result of the “flipped” data. For redundancy reasons we therefore only report the correlations for the “flipped” GM segments.

We also tested for a positive or negative relationship between each clinical feature (i.e., CRPS duration, current pain, pain experienced over the last month) and all GM or WM voxels using the SPM 8 regression analysis. Here we found no significant GM or WM clusters in none of the 6 regression analyses (see Methods for further details), even when applying a non-significant cluster threshold of p = 0.001, uncorrected.

## Discussion

In the present study we investigated possible alterations of gray and white matter brain structure in patients suffering from CRPS type I as compared to age and gender matched healthy controls. The main finding of this study was an increase in gray matter density in CRPS patients in one single significant cluster of voxels in the DMPFC, which in previous studies was shown to be involved in coding emotional correlates of pain [28], [29]. Using a region-of-interest based analysis we additionally found an increase in gray matter density located in the primary motor cortex (i.e., M1) contralateral to the CRPS-affected limb which was inversely related to decreased white matter density within the ipsilateral internal capsule possibly indicating compensatory mechanisms. However, we could not find a relationship between any clinical features and these structural brain alterations. Thus, we can only speculate on their origin.

### Structural alterations in the M1 and internal capsule

Next to the analysis of CRPS-related gray matter changes across the whole brain, we were specifically interested in CRPS-related influences on the S1 and the M1 representations of the affected limb. In previous functional brain imaging studies, we [13]–[16] and others ([11], [12], see [30] for a review and meta-analysis on the S1 function in CRPS) observed functional alterations of the cortical representation of the CRPS-affected limb on the postcentral and the precentral gyrus.

Here, we show an increase of gray matter density contralateral to the CRPS-affected limb, which was not located on the postcentral gyrus (i.e., anatomical site of the S1), but more anteriorly on the precentral gyrus, known as the anatomical site of the M1. The lack of gray matter alterations in the S1 agrees with many previous studies on brain structure in many different chronic pain syndromes. In contrast to healthy individuals receiving painful stimuli over several days, chronic pain patients seem not to develop an increase in gray matter density in contralateral somatosensory areas which suggests that these brain structures are not involved in chronic pain processes [31]. A possible explanation for these differences between healthy individuals and chronic pain patients is, that the pain experience in patients suffering from chronic pain is mostly driven by the brain itself and that the afferent noxious input is no longer of great relevance for the pain experience.

Gray matter density in the M1 did not relate to any clinical features, such as pain intensity or duration of CRPS. But we found a negative correlation between increased gray matter density in the M1 and decreased white matter density in the internal capsule; known to contain the descending projections from the M1 to the brainstem. Regarding the impact of CRPS on motor function, previous fMRI studies investigated cortical hemodynamic responses to simple finger tapping [32]. The M1 and the supplementary motor cortex (SMA) showed increased hemodynamic activation, which correlated with the individual extend of motor dysfunction. This amplified activation may over time consolidate to brain structure leading to the increased gray matter density we observed in the present study (see Fig. 2 and 3). The inverse relationship between the increased gray matter density in the M1 and the decreased white matter density in the internal capsule (see Fig. 3) possibly supports compensating for the amplified activation in the M1.

### Structural alterations in the DMPFC

The main finding of the current study was the increased gray matter structure we found in the DMPFC which is known to be implicated in a wide range of social and cognitive processes such as regulating emotional behavior [33], abusing trust to maximize financial reward [34], signaling negative outcomes in the context of risk aversion [35], or mediating empathic responses to others’ suffering, with the strength of its activity predicting the support offered to the victims [36], [37].

In the context of pain, the DMPFC appears to be crucially involved in coding the emotional correlates of pain anticipation [28], as well as the suffering from pain (see e.g., [29]). Since we found no relationship between any clinical features, such as pain intensity, and gray matter density in the DMPFC, we can only speculate on whether pain, the suffering from it, or the combination even with other processes is linked to the structural alterations of the DMPFC. One reason for the missing link between brain structure and pain intensity might be the variability of CRPS pain over time which affects the retrospective evaluations leading to an imprecise pain rating. A more suitable explanation for the missing correlation is the assumption that structural alterations must have developed over a longer time period than the one month that we captured with our pain intensity ratings. Nevertheless, based on its general emotional implementation, the DMPFC could be prone to coding the emotional aspects of pain, such as pain-related suffering or threat (see [38] for a meta-analysis), instead of pain intensity per se. Since we in the present study did not systematically assess emotional aspects of pain, future research will be needed to distinguish these aspects in more detail.

Regarding its connectivity, the DMPFC was identified as a crucial hub in networks generating several emotional states. Many studies showed that the DMPFC is consistently co-activated with the hypothalamus and the periaqueductal gray (PAG), that both contribute to emotional modulation (see [39] for a meta-analysis). These evidences on functional connectivity convincingly agree with anatomical studies in monkey that identified unidirectional projection between the DMPFC and the PAG [40], [41], as well as between the DMPFC and the hypothalamus [42], [43]. Together these findings suggest that the DMPFC is part of an appraisal system involved in the cognitive generation of emotions [39]. Besides modulation of emotional states, hypothalamus and the PAG are also known to underpin modulation of pain which renders the DMPFC as a possible candidate region for mediating analgesia [44], [45]. This speculation is supported by recent neuroimaging findings identifying the DMPFC as a salient component of acupuncture analgesia probably contributing to top-down modulation of central pain networks [46].

### Comparison of present with previous studies on brain structure in CRPS

The present findings do not obviously agree with previous studies on altered brain structure of CRPS patients [20]. Although applying same techniques to study gray matter structure (i.e., VBM on T1-weighted MRI scans), Geha and colleagues observed CRPS related gray matter alterations in the insula cortex, the nucleus accumbens, and the VMPFC [20]. Like the DMPFC, also the VMPFC is well known for its involvement in emotional processes, suggesting that the debilitating chronic pain with its emotional burden may alter the processing in these brain regions which over time consolidates to brain structure. However, contrarily to the findings of Geha and colleagues, we did not observe a reduced but increased gray matter density, and not in the VMPFC, but in the DMPFC as another important hub of the emotional brain network. In our study, alterations in gray matter density however did not relate to any clinical features remaining its clinical implementation speculative. But the differences between previous [20] and our findings might be explained by differences between the two cohorts. We recruited only patients with CRPS type I to exclude possible interfering influences on cortical reorganization due to peripheral nerve damage (see e.g. [21]). But more importantly, Geha and colleagues [20] examined patients with a much longer CRPS duration (up to 14 years as compared to 5 years in our cohort) and clinical features of CRPS can substantially vary over time (see e.g. [5], [47]).

Our findings of an increased gray matter density in the DMPFC and the M1 also do not resemble previous findings in other chronic pain syndromes [48]–[52], that seem to be rather characterized by a decrease instead of an increase of gray matter density in pain transmitting brain structures. The pattern of decreased gray matter density is different for each of these pain syndromes but seems to overlap in certain brain regions involved in the experience and anticipation of pain such as the cingulate cortex, the insula, and the orbitofrontal cortex [31]. Healthy individuals sensitized to pain over several days of daily painful stimulations also showed a decreased gray matter density in the anterior cingulate cortex, the insula and the frontal cortex, whereas pain habituaters either showed no structural alterations [53], or an increase of gray matter density in mid-cingulate and somatosensory cortex [31]. Chronic pain patients generally seem to have a reduced ability to habituate to pain, which might relate to the decreased gray matter density of pain transmitting brain regions, possibly reflecting a constraint of the antinociceptive system [31]. Based on these studies in chronic pain syndromes and healthy individuals, one might speculate that the discrepancy between the present (i.e., increased gray matter density) and previous findings in CRPS (i.e., decreased gray matter density, see [20]) may rely on the differences in the duration of the syndrome possibly reflecting the transition to an advancing reduction in pain habituation. Additionally, this discrepancy supports the assumption of a multifactorial disease with different factors and symptoms differentially consolidate to brain structure.
